# Supplementary material for: The challenge of intracellular antibiotic accumulation, a function of fluoroquinolone influx versus bacterial efflux
Source: Commun Biol. 2020 Apr 28;3:198. doi: 10.1038/s42003-020-0929-x (PMC7189378; doi:10.1038/s42003-020-0929-x)
Supplement: Supplementary file 2 — Description of Additional Supplementary Files [file 42003_2020_929_MOESM2_ESM.pdf]

Description of Additional Supplementary Files

File Name: **Supplementary Data 1**
Description: This document contains the crude results used for the main figures. This is explained in the section "Statistics and Reproducibility".
