# Supplementary material for: The challenge of intracellular antibiotic accumulation, a function of fluoroquinolone influx versus bacterial efflux
Source: Commun Biol. 2020 Apr 28;3:198. doi: 10.1038/s42003-020-0929-x (PMC7189378; doi:10.1038/s42003-020-0929-x)
Supplement: Supplementary file 3 — Supplemental Material [file 42003_2020_929_MOESM3_ESM.docx]

**Supplementary Information**

**Supplementary Figure 1.**

**
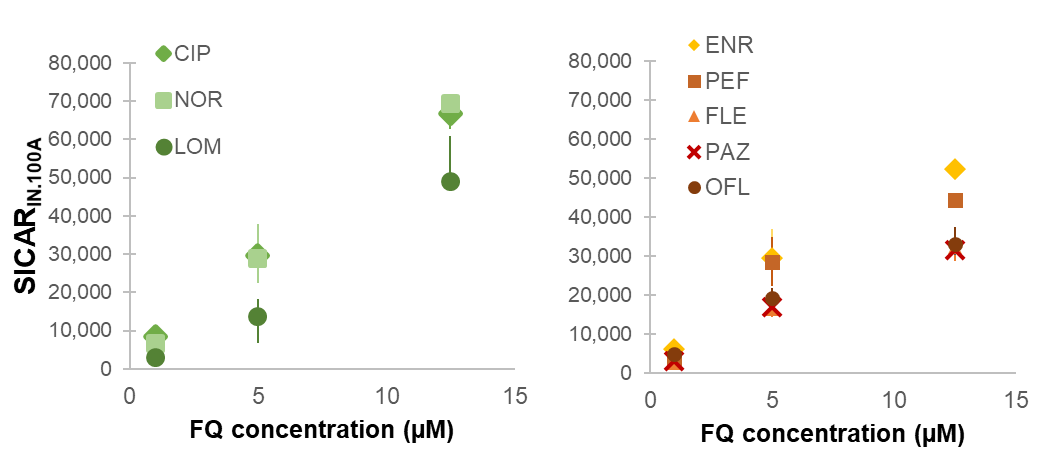
**

**Supplementary Figure 1. Effect of concentrations on SICAR_IN.100A._**

Bacterial suspensions of AG100A cells (AcrAB deficient mutant) were incubated with a given FQ at 1 µM, 5 µM or 12.5 µM. SICAR_IN.100A_ were determined from the accumulated concentration of FQs measured in AG100A with spectrofluorimetry. SICAR_IN.100A_ of FQs with no saturation effect are shown on the left panel and SICAR_IN.100A_ with a slight saturated effect are shown on the right panel.

**Supplementary Figure 2.**

**
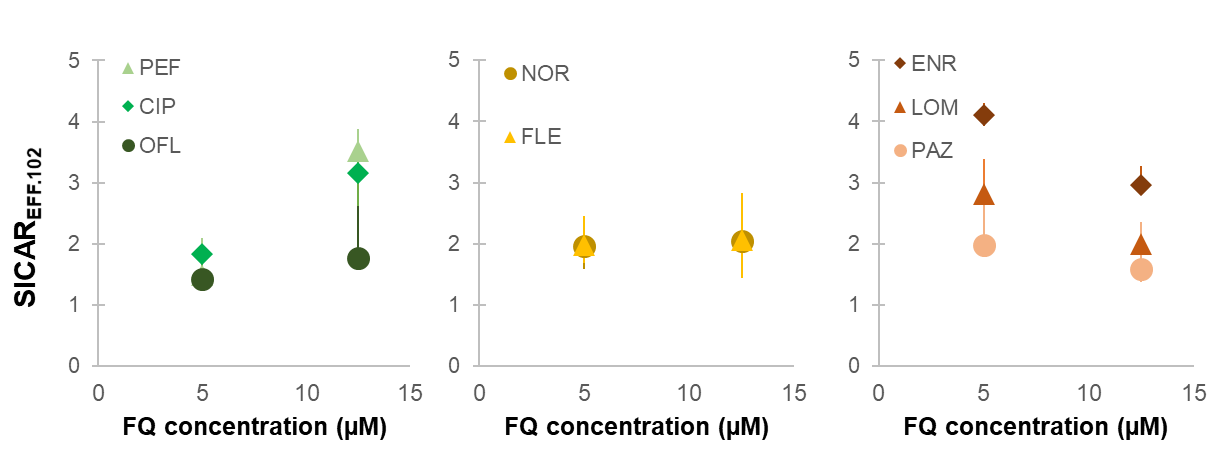
**

**Supplementary Figure 2. Effect of concentrations on SICAR_EFF.102._**

Bacterial suspensions of AG100A (AcrAB deficient mutant) and AG102 cells (overexpressing AcrAB) were incubated with a given FQ at 5 µM or 12.5 µM. SICAR_EFF.102_ were calculated as the ratio of the accumulated concentration in AG100A to the accumulated concentration in AG102 measured with spectrofluorimetry. SICAR_EFF.102_ that increase with increasing concentrations are shown on the left panel, FQs with constant SICAR_EFF.102_ are shown on the middle panel, and SICAR_EFF.102_ that decrease with increasing concentration are shown on the right panel.

**Supplementary Figure 3.**

**Supplementary Figure 3. Efflux susceptibility of FQs measured with microspectrofluorimetry on individual cells.** Accumulation of ENR, FLE and NOR in AG102 cells (overexpressing AcrAB) with (upper panel) and without CCCP (lower panel) measured by DUV microspectrofluorimetry. CCCP was used as an efflux blocker, which collapses the energy-driven force needed by the efflux pump. Cells were incubated for 5 min at 37°C with a given FQ with or without 10 µM CCCP. Control samples were cultured using the same procedure but without adding the drugs in the incubation step. Cell pellets were resuspended in buffer and sandwiched between two quartz coverslips before the analysis by DUV fluorescence imaging (see Experimental procedures). Data are represented by violin plots, a representation similar to box plots that allows to show the probability density of the data at different values. The plotted data correspond to the drug normalized fluorescence intensities measured in 173/483/748 and 224/375/643 individual cells for ENR/FLE/NOR, with and without CCCP respectively.

It can be seen that in the case of AG102 with CCCP, the measured accumulation of ENR is more pronounced compared to the other two FQs, whereas, in the case of AG102 strain without the addition of CCCP, the accumulation ratios are comparable. This result additionally demonstrates that the efflux susceptibility of ENR is higher than the susceptibilities of FLE and NOR fluoroquinolones.

**Supplementary Figure 4.**

**
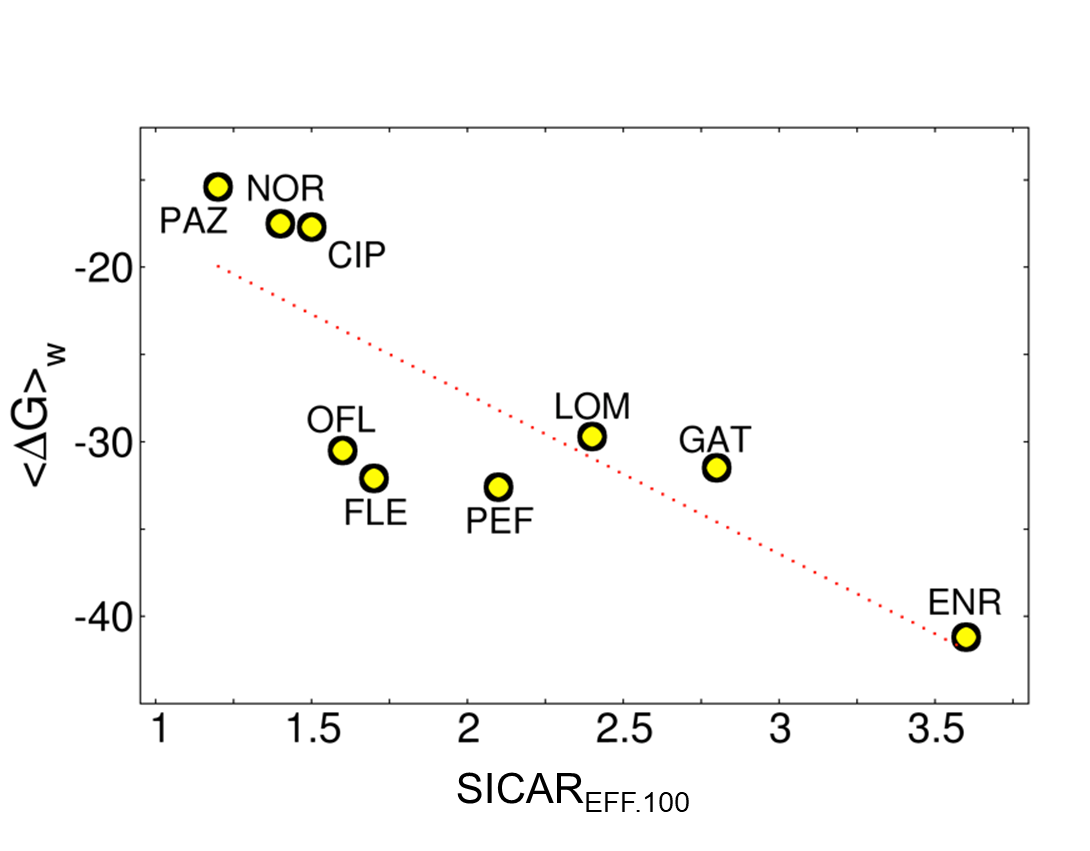
**

**Supplementary Figure 4. Correlation plot between SICAR_EFF.100_ data measured by spectrofluorimetry and weighted average binding free energies (<ΔG’>_w_) calculated from MD simulations.** SICAR_EFF.100_ are the ratio of the accumulated concentration of FQs in the efflux deficient mutant AG100A to the accumulated concentration in the wild-type strain AG100 (See Figure 4A). <ΔG’>_w_ represents the weighted average binding affinity of each FQ for the DP_T_ of AcrB (See Figure 5). The linear regression equation best fitting the experimental data is <ΔG’>_w_ = -8.9654 – 9.1536 x SICAR_EFF.100_; the corresponding Pearson’s correlation coefficient R^2^ is 0.8190.

**Supplementary Figure 5.**


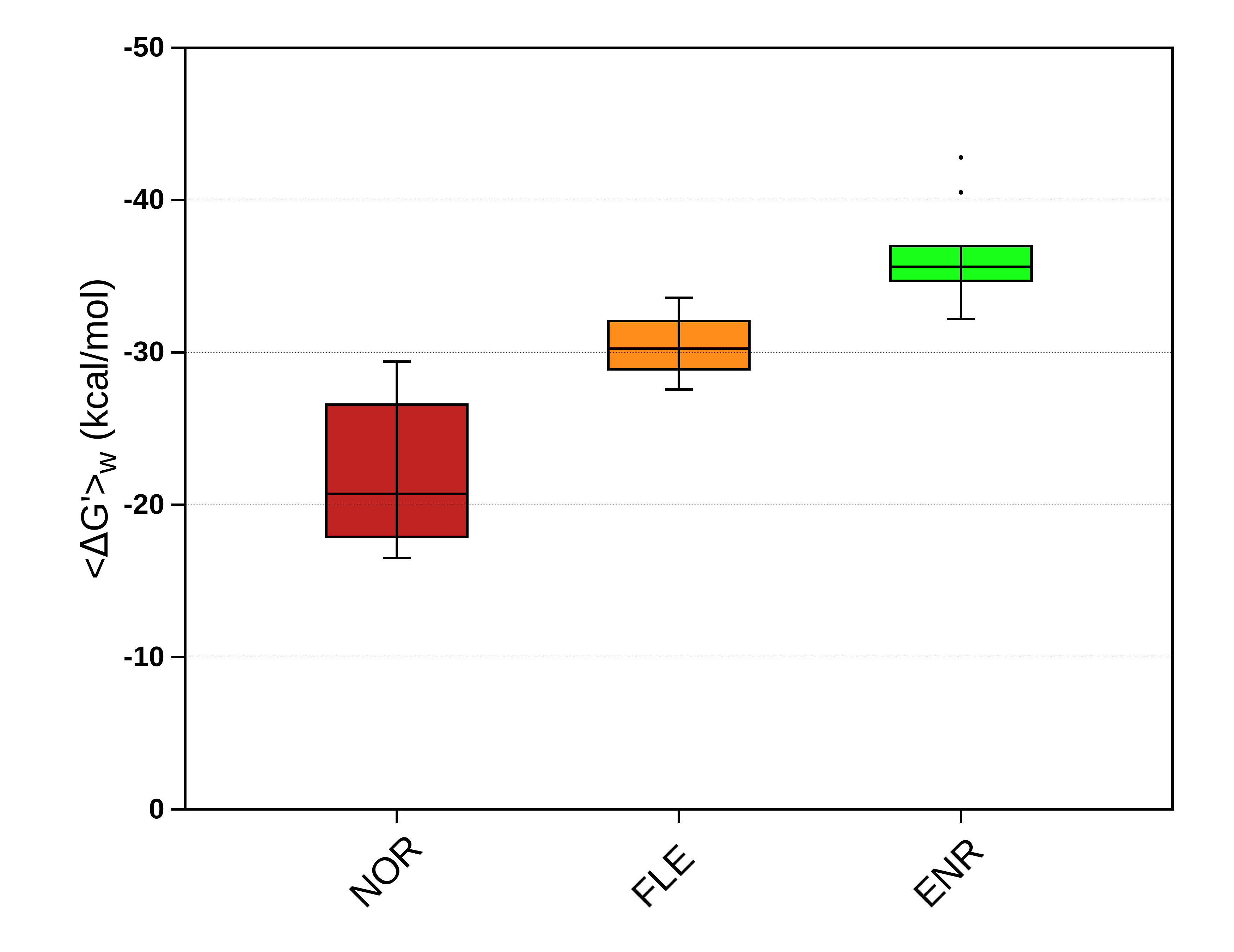


**Supplementary Figure 5. Weighted average free energy of binding (<ΔG’>_w_) for NOR, FLE, and ENR.** The reported values were extracted from ten MD simulations performed for each compound by considering conformational clusters populated by at least 10 % of the total simulation time (data reported in Table S4).

**Supplementary Figure 6.**


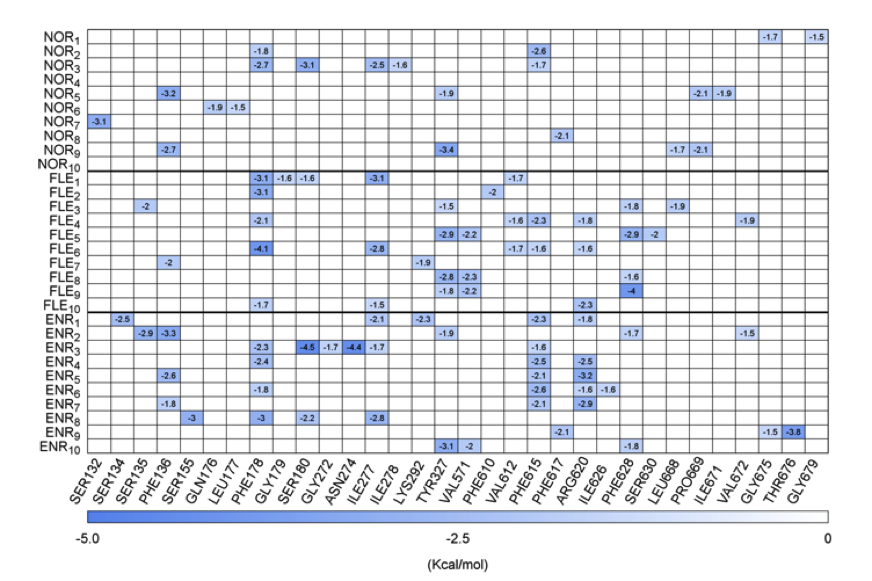


**Supplementary Figure 6. Per-residue contributions to the ΔG’ calculated with the Molecular Mechanics/Generalized Born Surface Area method.** The reported data correspond to the 10 simulations performed for NOR, FLE, and ENR in the DP_T_ of AcrB for the most populated cluster of each simulation. Only protein residues contributing more than -1.5 kcal mol^-1^ to the total free energy of binding are shown.

**Supplementary Table 1.**

|  |  | **MIC (µM)** |  |  | **MIC (µM)** |  |  | **DEK (µM)** |  |
| --- | --- | --- | --- | --- | --- | --- | --- | --- | --- |
|  | **AG100** | **AG100A** | **AG102** | **AG100 + PAβN** | **AG100A + PAβN** | **AG102**  **+ PAβN** | **AG100** | **AG100A** | **AG102** |
| **NAL** | 16 | 8 | 64 | 4 | 2-4 | 4 | 16 | 4 | 64 |
| **FLU** | 4 | 0.5 | 16 | 0.5 | 0.25 | 0.5 | 2 | 0.25 | 8 |
| **CIP** | 0.0625 | 0.016 | 0.5 | 0.031 | 0.008 | 0.25 | 0.0625 | 0.031 | 0.5 |
| **ENR** | 0.0625 | 0.031 | 0.5 | 0.016 | 0.031 | 0.031 | 0.0625 | 0.031 | 0.5 |
| **ENO** | 0.5 | 0.25 | 2 | 0.5 | 0.125 | 1 | 0.5 | 0.125 | 2 |
| **FLE** | 0.125 | 0.06 | 1 | 0.0625 | 0.062 | 0.25 | 0.125 | 0.0625 | 1 |
| **LOM** | 0.5 | 0.125 | 2 | 0.125 | 0.062 | 0.25 | 0.125 | 0.0625 | 1 |
| **NOR** | 0.5 | 0.125 | 2 | 0.5 | 0.062 | 2 | 0.25 | 0.0625 | 2 |
| **NAD** | 0.5 | 0.016 | 2 | 0.016 | 0.004 | 0.062 | 1 | 0.016 | 2 |
| **OFL** | 0.25 | 0.031 | 1 | 0.062 | 0.031 | 0.25 | 0.125 | 0.0625 | 1 |
| **PEF** | 0.25 | 0.125 | 2 | 0.062 | 0.062 | 0.25 | 0.25 | 0.031 | 0.5 |
| **BAL** | 0.5 | 0.125 | 2 | 0.125 | 0.062 | 0.5 | 0.5 | 0.031 | 2 |
| **LEV** | 0.125 | 0.016 | 1 | 0.031 | 0.031 | 0.125 | 0.125 | 0.016 | 0.5 |
| **PAZ** | 0.125 | 0.06 | 0.5 | 0.062 | 0.031 | 0.125 | 0.25 | 0.031 | 0.5 |
| **SPA** | 0.031 | 0.008 | 0.25 | 0.008 | 0.008 | 0.016 | 0.031 | 0.004 | 0.25 |
| **TOS** | 0.031 | 0.008 | 0.25 | 0.008 | 0.008 | 0.016 | 0.031 | 0.016 | 0.5 |
| **CLI** | 0.031 | 0.016 | 0.125 | 0.016 | 0.008 | 0.016 | 0.031 | 0.016 | 0.25 |
| **GEM** | 0.031 | 0.008 | 0.25 | 0.016 | 0.004 | 0.0625 | 0.031 | 0.016 | 0.25 |
| **MOX** | 0.125 | 0.031 | 1 | 0.031 | 0.031 | 0.0625 | 0.25 | 0.0625 | 1 |
| **GAT** | 0.125 | 0.031 | 0.5 | 0.031 | 0.016 | 0.125 | 0.0625 | 0.016 | 0.5 |
| **TRO** | 0.0625 | 0.031 | 0.25 | 0.008 | 0.008 | 0.016 | 0.0625 | 0.0625 | 0.5 |
| PAβN; *phenyl-arginine-ß-naphthylamide used at 20 mg l^-1^; MIC: Minimal inhibitory concentration; DEK: Dose for early killing is defined as the lowest concentration inducing a reduction in the growth of bacteria determined with the resazurin-based viability assay.* | | | | | | | | | |

**Supplementary Table 1. MIC and DEK of the various FQs investigated.**

**Supplementary Table 2.**

| PDB IDs of X-ray structures | 2DHH, 2J8S, 2GIF, 3W9H, 4DX5, 4DX7, 4U8V, 4U8Y, 4U95, 4U96 |
| --- | --- |
| Deep Binding Pocket (DP) | SER46, GLN89, SER128, GLU130, SER134, PHE136, VAL139, GLN176, LEU177, PHE178, GLY179, GLY180, GLU273, ASN274, ASP276, ILE277, TYR327, MET573, PHE610, VAL612, PHE615, PHE617, ARG620, PHE628 |
| Switch Loop | GLY616, PHE617, ALA618, GLY619 |
| Bottom Loop | ALA670 – THR676 |
| Exit Gate (EG) | GLN124, GLN125, TYR758, LYS770 |

**Supplementary Table 2. List of the PDB ID codes of the X-ray structures of AcrB used for molecular docking calculations, and the residue composition of AcrB regions shown in Figure 6b**.

Protein residues identifying the hydrophobic trap (HP-trap) within the DP are underlined.

Supplementary Table 3.

| **Trajectory** | **Pop (%)** | **ΔG’** | **<ΔG’>_w_** |
| --- | --- | --- | --- |
| CIP | 55.7 | -26.0 (3.8) | -18.3 (3.9) |
|  | 30.4 | -17.7 (4.0) |  |
|  | 13.4 | -19.9 (6.3) |  |
|  | 63.2 | -12.7 (3.2) |  |
|  | 15.6 | -13.2 (4.4) |  |
| ENR | 99.1 | -47.7 (5.3) | -41.1 (5.4) |
|  | 100.0 | -34.6 (5.4) |  |
| FLE | 91.7 | -29.3 (4.0) | -32.2 (4.3) |
|  | 100.0 | -34.8 (4.5) |  |
| GAT | 90.7 | -34.0 (5.7) | -31.4 (4.7) |
|  | 100.0 | -29.0 (3.5) |  |
| LOM | 51.2 | -34.4 (5.2) | -29.7 (4.6) |
|  | 48.2 | -25.0 (5.9) |  |
|  | 71.9 | -28.8 (3.6) |  |
|  | 17.2 | -37.0 (3.4) |  |
|  | 10.9 | -22.8 (2.6) |  |
| NOR | 60.4 | -14.5 (3.9) | -17.5 (4.0) |
|  | 27.5 | -15.4 (4.3) |  |
|  | 11.9 | -23.4 (4.9) |  |
|  | 100.0 | -19.2 (3.8) |  |
| OFL | 100 | -32.1 (3.7) | -30.5 (3.7) |
|  | 51.9 | -30.5 (3.1) |  |
|  | 40.3 | -26.6 (4.4) |  |
| PAZ | 99.4 | -17.3 (4.6) | -15.4 (4.7) |
|  | 63.5 | -13.2 (4.2) |  |
|  | 20.3 | -13.9 (6.4) |  |
|  | 16.1 | -14.4 (4.4) |  |
| PEF | 83.5 | -26.9 (3.1) | -32.5 (5.1) |
|  | 16.5 | -33.1 (2.8) |  |
|  | 66.0 | -38.0 (6.5) |  |
|  | 29.0 | -35.8 (6.7) |  |

Supplementary Table 3. Solvation free energy of binding calculated with the MM/GBSA method (ΔG’, expressed in kcal mol^-1^) on the structural clusters extracted from MD trajectories for each AcrB-FQ complex along with their population (%).

Last column reports the weighted average free energy of binding (<ΔG’>w) for each compound. Standard deviations are reported within parenthesis.

Supplementary Table 4.

| **Trajectory** | **Pop (%)** | **avg 1st hyd shell** | **ΔG’** | **<ΔG’>_w_** | **<ΔG’>_w_ TOT** |
| --- | --- | --- | --- | --- | --- |
| NOR_1_ c1 | 59.8 | 17.9 (3.2) | -24.7 (3.6) | -24.7(3.9) | -22.0 (3.8) |
| NOR_1_ c2 | 28.8 | 13.8 (2.9) | -24.1 (4.2) |  |  |
| NOR_1_ c3 | 11.1 | 13.7 (2.8) | -26.4 (4.4) |  |  |
| NOR_2_ c1 | 94.0 | 20.0 (2.6) | -17.9 (3.2) | -17.9 (3.2) |  |
| NOR_3_ c1 | 100.0 | 14.2 (2.2) | -27.5 (3.2) | -27.5 (3.2) |  |
| NOR_4_ c1 | 63.3 | 20.2 (3.0) | -20.3 (5.0) | -19.4 (4.9) |  |
| NOR_4_ c2 | 28.2 | 22.1 (3.5) | -17.4 (4.6) |  |  |
| NOR_5_ c1 | 61.3 | 16.3 (2.7) | -26.5 (3.5) | -26.6 (3.6) |  |
| NOR_5_ c2 | 38.1 | 13.0 (3.6) | -26.7 (3.7) |  |  |
| NOR_6_ c1 | 77.9 | 19.5 (2.5) | -21.2 (2.5) | -20.9 (2.7) |  |
| NOR_6_ c2 | 20.9 | 20.8 (4.0) | -19.6 (3.3) |  |  |
| NOR_7_ c1 | 79.1 | 17.5 (2.4) | -22.0 (3.5) | -20.6 (3.5) |  |
| NOR_7_ c2 | 18.7 | 22.8 (3.2) | -14.7 (3.3) |  |  |
| NOR_8_ c1 | 36.6 | 24.7 (3.7) | -14.4 (4.1) | -16.1 (4.2) |  |
| NOR_8_ c2 | 35.6 | 22.1 (3.4) | -14.6 (3.8) |  |  |
| NOR_8_ c3 | 23.3 | 20.4 (3.6) | -21.2 (4.9) |  |  |
| NOR_9_ c1 | 99.9 | 10.5 (2.8) | -29.4 (4.7) | -29.4 (4.7) |  |
| NOR_10_ c1 | 100.0 | 17.6 (3.0) | -16.6 (3.8) | -16.6 (3.8) |  |
| FLE_1_ c1 | 90.4 | 13.6 (2.6) | -28.9 (5.1) | -28.9 (5.1) | -30.3 (4.4) |
| FLE_2_ c1 | 59.9 | 10.7 (3.6) | -33.3 (4.1) | -32.1 (3.9) |  |
| FLE_2_ c2 | 11.4 | 13.0 (3.4) | -31.8 (3.1) |  |  |
| FLE_2_ c3 | 11.2 | 15.1 (3.3) | -25.7 (3.2) |  |  |
| FLE_3_ c1 | 60.6 | 8.3 (2.4) | -35.4 (5.2) | -33.6 (5.0) |  |
| FLE_3_ c2 | 39.2 | 11.1 (2.2) | -30.8 (4.7) |  |  |
| FLE_4_ c1 | 93.8 | 9.7 (2.2) | -32.9 (3.7) | -32.9 (3.7) |  |
| FLE_5_ c1 | 100.0 | 10.9 (3.0) | -30.5 (3.2) | -30.5 (3.2) |  |
| FLE_6_ c1 | 31.6 | 13.2 (2.7) | -29.8 (3.9) | -27.6 (4.7) |  |
| FLE_6_ c2 | 29.6 | 14.9 (3.6) | -23.7 (4.7) |  |  |
| FLE_6_ c3 | 28.7 | 14.1 (2.9) | -29.1 (5.4) |  |  |
| FLE_7_ c1 | 86.7 | 16.5 (3.6) | -27.6 (5.4) | -27.6 (5.4) |  |
| FLE_8_ c1 | 71.4 | 13.1 (2.6) | -28.9 (4.4) | -29.1 (4.1) |  |
| FLE_8_ c2 | 28.6 | 11.9 (2.4) | -29.5 (3.2) |  |  |
| FLE_9_ c1 | 100.0 | 7.8 (2.7) | -30.0 (4.3) | -30.0 (4.3) |  |
| FLE_10_ c1 | 87.6 | 10.5 (2.2) | -30.8 (4.1) | -30.6 (4.1) |  |
| FLE_10_ c2 | 11.1 | 9.4 (2.1) | -29.0 (4.2) |  |  |
| ENR_1_ c1 | 100.0 | 16.6 (2.5) | -35.0 (4.4) | -35.0 (4.4) | -36.4 (4.5) |
| ENR_2_ c1 | 100.0 | 7.4 (2.5) | -42.8 (5.8) | -42.8 (5.8) |  |
| ENR_3_ c1 | 96.3 | 10.5 (2.3) | -40.5 (5.6) | -40.5 (5.6) |  |
| ENR_4_ c1 | 92.4 | 12.0 (2.5) | -33.9 (3.3) | -33.9 (3.3) |  |
| ENR_5_ c1 | 52.7 | 9.8 (2.2) | -36.9 (3.7) | -36.9 (3.8) |  |
| ENR_5_ c2 | 47.3 | 9.1 (2.1) | -37.0 (3.9) |  |  |
| ENR_6_ c1 | 100.0 | 10.7 (2.3) | -37.0 (3.7) | -37.0 (3.7) |  |
| ENR_7_ c1 | 99.9 | 14.6 (3.0) | -32.2 (5.5) | -32.2 (5.5) |  |
| ENR_8_ c1 | 95.7 | 14.3 (2.8) | -34.7 (4.4) | -34.7 (4.4) |  |
| ENR_9_ c1 | 38.4 | 9.6 (2.5) | -39.0 (3.5) | -34.7 (4.1) |  |
| ENR_9_ c2 | 17.6 | 15.2 (3.1) | -28.3 (3.8) |  |  |
| ENR_9_ c3 | 13.0 | 13.0 (2.9) | -33.2 (5.3) |  |  |
| ENR_9_ c4 | 11.9 | 10.9 (2.4) | -32.2 (4.7) |  |  |
| ENR_10_ c1 | 79.4 | 8.3 (1.8) | -35.7 (3.3) | -36.3 (3.3) |  |
| ENR_10_ c2 | 15.8 | 5.6 (2.3) | -39.1 (3.0) |  |  |

Supplementary Table 4. Total free energy of binding calculated with the MM/GBSA method (ΔG’, kcal mol^-1^) for the cluster trajectories identified for each simulation along with their population (%) and average number of water molecules located in the first solvation shell (cut-off distance 3.5 Å).

The fifth column reports the weighted average free energy of binding (<ΔG’>w) for each MD simulation, while last column shows the total weighted average free energy of binding (<ΔG’>w TOT) for each compound. Standard deviations are reported within parenthesis.
